# Tempo and mode of regulatory evolution in *Drosophila*

**DOI:** 10.1101/gr.163014.113

**Published:** 2014-05

**Authors:** Joseph D. Coolon, C. Joel McManus, Kraig R. Stevenson, Brenton R. Graveley, Patricia J. Wittkopp

**Affiliations:** 1 University of Michigan, Department of Ecology and Evolutionary Biology, Ann Arbor, Michigan 48109, USA;; 2 Carnegie Mellon University, Department of Biological Sciences, Pittsburgh, Pennsylvania 15213, USA;; 3 University of Connecticut Health Center, Institute for Systems Genomics, Department of Genetics and Developmental Biology, Farmington, Connecticut 06030, USA;; 4 University of Michigan, Department of Computational Medicine and Bioinformatics, Ann Arbor, Michigan 48109, USA;; 5 University of Michigan, Department of Molecular, Cellular, and Developmental Biology, Ann Arbor, Michigan 48109, USA

## Abstract

Genetic changes affecting gene expression contribute to phenotypic divergence; thus, understanding how regulatory networks controlling gene expression change over time is critical for understanding evolution. Prior studies of expression differences within and between species have identified properties of regulatory divergence, but technical and biological differences among these studies make it difficult to assess the generality of these properties or to understand how regulatory changes accumulate with divergence time. Here, we address these issues by comparing gene expression among strains and species of *Drosophila* with a range of divergence times and use F_1_ hybrids to examine inheritance patterns and disentangle *cis*- and *trans*-regulatory changes. We find that the fixation of compensatory changes has caused the regulation of gene expression to diverge more rapidly than gene expression itself. Specifically, we observed that the proportion of genes with evidence of *cis*-regulatory divergence has increased more rapidly with divergence time than the proportion of genes with evidence of expression differences. Surprisingly, the amount of expression divergence explained by *cis*-regulatory changes did not increase steadily with divergence time, as was previously proposed. Rather, one species (*Drosophila sechellia*) showed an excess of *cis*-regulatory divergence that we argue most likely resulted from positive selection in this lineage. Taken together, this work reveals not only the rate at which gene expression evolves, but also the molecular and evolutionary mechanisms responsible for this evolution.

Understanding the relationship between tempo (the rate at which a trait evolves) and mode (the manner in which a trait evolves) is essential for understanding the evolutionary process (Simpson 1944). This is true not only for organismal phenotypes, but also for the molecular phenotypes that produce organismal traits. Gene expression is one such molecular phenotype (Gordon and Ruvinsky 2012); it is essential for organismal form, fitness, and function, and frequently varies within and between species. Comparative studies using genomic surveys of gene expression in yeast (Busby et al. 2011), *Drosophila* (Rifkin et al. 2003), and mammalian species (Brawand et al. 2011) with a range of divergence times have provided insight into the tempo of gene expression evolution, but the mode and its relationship to tempo remain less well understood.

Elucidating the mode of gene expression evolution includes identifying the types of regulatory changes that have evolved as well as how interactions among divergent regulatory alleles affect gene expression. F_1_ hybrids, in which divergent regulatory alleles interact in the same cellular environment, can be used to investigate these issues. Allele-specific expression in F_1_ hybrids separates the effects of *cis*- and *trans*-regulatory changes affecting a gene’s expression by providing a readout of relative *cis*-regulatory activity in a common *trans*-regulatory environment (Cowles et al. 2002). Expression differences between genotypes not attributed to *cis*-regulatory changes are inferred to be caused by *trans*-regulatory divergence (Wittkopp et al. 2004). In addition, the net effects of interactions among divergent regulatory alleles are revealed by comparing levels of total expression in F_1_ hybrids to parental genotypes.

This approach was initially used to separate *cis*- and *trans*-regulatory effects of divergence affecting expression of dozens of genes. These studies suggested that (1) *cis*-regulatory changes are more common than *trans*-regulatory changes between species (Wittkopp et al. 2004); (2) genes with *cis*- and *trans*-acting changes favoring expression of opposite alleles are more likely than other types of changes to cause misexpression in F_1_ hybrids (Landry et al. 2005); (3) environmental factors modulate relative *cis*-regulatory activity (de Meaux et al. 2006); (4) *cis*-regulatory variation is abundant in natural populations (Osada et al. 2006; Genissel et al. 2007; Campbell et al. 2008; Gruber and Long 2009); and (5) the amount of expression divergence attributable to *cis*-acting changes is greater between than within species (Wittkopp et al. 2008).

More recently, microarrays and RNA-seq have been used to extend these analyses to the genomic scale (Wang et al. 2008; Graze et al. 2009; Tirosh et al. 2009; Zhang and Borevitz 2009; Fontanillas et al. 2010; McManus et al. 2010; He et al. 2012; Shi et al. 2012; Coolon and Wittkopp 2013; Levy et al. 2013; Schaefke et al. 2013). In some cases, relationships seen in the smaller scale studies were replicated. For example, *cis*- and *trans*-regulatory changes with effects in opposite directions were overrepresented among misexpressed genes (Tirosh et al. 2009; McManus et al. 2010; Schaefke et al. 2013) and *cis*-regulatory changes explained more of the expression differences between than within species (Tirosh et al. 2009; Emerson et al. 2010). Other observations, such as the relative proportion of genes with evidence of *cis*- and/or *trans*-regulatory changes, were much more variable among studies. Finally, novel patterns, such as the relationship between dominance and *cis*/*trans*-regulatory changes (Lemos et al. 2008; McManus et al. 2010) and the frequency of compensatory *cis*- and *trans*-regulatory variants (Tirosh et al. 2009; Goncalves et al. 2012; Shi et al. 2012), were identified.

Despite this growing collection of case studies examining the types of changes responsible for expression differences within and/or between particular pairs of species, the use of different organisms (flies, yeast, plants, and mice), techniques (pyrosequencing, microarrays, RNA-seq), and analysis methods (linear models, exact tests, and Bayesian approaches) among these studies precludes the type of meta-analysis needed to determine how the mode of regulatory evolution changes with divergence time and to robustly assess the generality of relationships reported in previous studies. To address these issues, we examined the tempo and mode of regulatory evolution in concert using strains and species of *Drosophila* with a range of divergence times.

## Results

### Experimental overview

**Figure 1.**
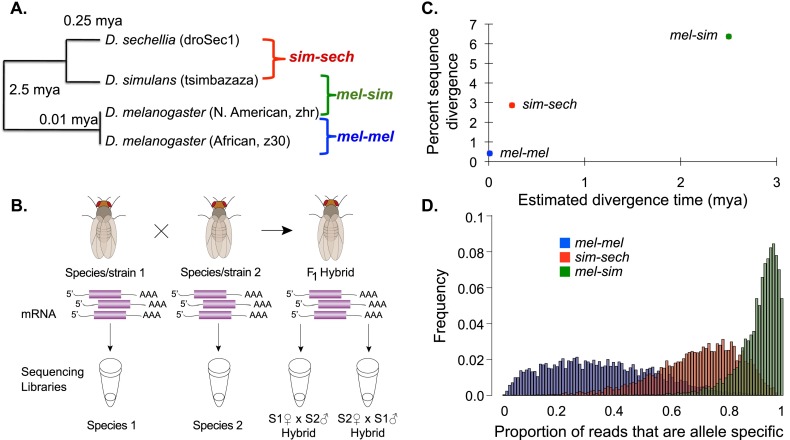
Studying regulatory evolution in the melanogaster group of *Drosophila*. (*A*) Phylogenetic relationships and estimated divergence times for the strains and species analyzed are shown. (*B*) Sequencing libraries for RNA-seq data were derived from mRNA isolated from each species and strain as well as F_1_ hybrids from reciprocal crosses, in which the maternal and paternal genotypes were reversed (e.g., S1 × S2 and S2 × S1). (*C*) The percent sequence divergence observed in the regions of the genome used to map RNA-seq reads (*y*-axis) is compared with published estimates of divergence time (*x*-axis). (*D*) The proportion of reads from each gene that is allele-specific is shown for the mel-mel (blue), sim-sech (red), and mel-sim (green) comparisons.

mRNA abundance was compared among (1) African and non-African strains of *Drosophila melanogaster* (mel-mel), which have been geographically isolated for ∼10,000 yr and show evidence of behavioral isolation (David and Capy 1988; Lachaise et al. 1988; Wu et al. 1995; Hollocher et al. 1997) and expression divergence (Hutter et al. 2008); (2) *D. simulans* and *D. sechellia* (sim-sech), which diverged ∼250,000 yr ago (Garrigan et al. 2012); and (3) *D. melanogaster* and *D. simulans* (mel-sim), which diverged ∼2.5 million yr ago (Fig. 1A; Cutter 2008). For each of these genotypes, we derived a strain-specific genome sequence and used RNA-seq to measure mRNA abundance (hereafter referred to as expression) in a pool of 20 adult female flies. Reciprocal crosses were performed for each of the three pairs of genotypes (mel-mel, sim-sech, and mel-sim), and RNA-seq was used to measure both total and allele-specific expression in pools of 20 female F_1_ hybrids from each cross (Fig. 1B). Sequence divergence observed in transcribed regions of these strains correlated with published estimates of divergence time (Fig. 1C) as well as the number of RNA-seq reads informative for allele-specific expression (Fig. 1D). Gene-specific and allele-specific read counts were used to investigate regulatory evolution as shown in Supplemental Figure S1.

### Quantifying gene expression levels

For each comparison (mel-mel, sim-sech, and mel-sim), RNA-seq reads from the two strains or species and their F_1_ hybrids were aligned to the relevant genomes and mapped to specific genes. Differences in sequencing depth among libraries (Supplemental Table S1) were eliminated by using random sampling without replacement to produce a data set with the same number of mapped reads for each sample. After excluding genes with fewer than 20 mapped reads in any sample (Supplemental Table S2), 7587 genes were deemed suitable for comparing total expression levels between all pairs of genotypes and their F_1_ hybrids (Data set 1), which is 83% of the genes classified as expressed in *D. melanogaster* adult females by modENCODE (Graveley et al. 2011).

**Figure 2.**
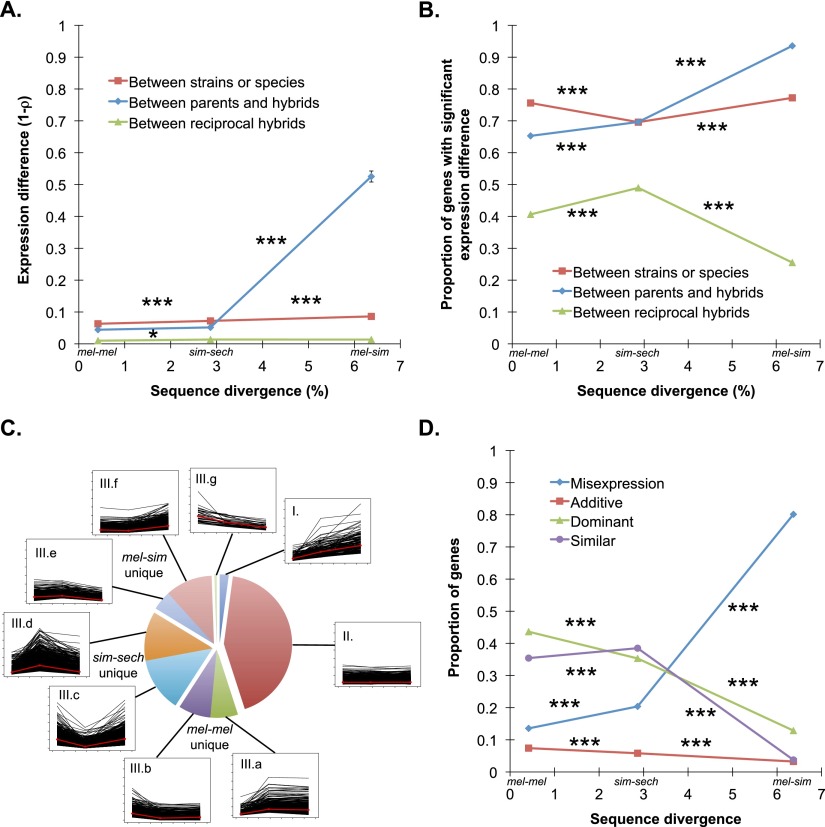
Expression divergence between genotypes and in F_1_ hybrids. (*A*) Overall expression divergence (1 − ρ) is shown for the mel-mel, sim-sech, and mel-sim comparisons in red, with the data used for these calculations shown in Supplemental Figure S5. Average differences in expression between F_1_ hybrids and each of the parental species are shown in blue, with the data used for these calculations shown in Supplemental Figure S7. Expression divergence between reciprocal F_1_ hybrids is included as a baseline in green, with the data used for these calculations shown in Supplemental Figure S3. In this and all other figures, results from each comparison are plotted using the genomic sequence divergence observed between the genotypes involved (Fig. 1C). (*B*) The proportion of genes showing evidence of significant expression differences between genotypes (red), the average proportion of genes showing significant expression differences between F_1_ hybrids and each parental species (blue), and the proportion of genes with significant expression differences between reciprocal F_1_ hybrid genotypes (green) are shown. (*C*) The line plots show expression differences for individual genes in the mel-mel, sim-sech, and mel-sim comparisons plotted according to divergence time, with the 7587 genes included in Data set 1 classified into nine groups depending on whether they showed increased, decreased, or similar expression differences between mel-mel and sim-sech and between sim-sech and mel-sim. The red line in each plot shows the median expression difference for genes in that class for each comparison. The pie chart shows the relative frequency of genes in each class. (*D*) The proportion of genes showing expression levels in F_1_ hybrids consistent with additive inheritance (red), dominant inheritance (green), misexpression (blue), or similar expression (purple) is shown for each comparison. Data used to calculate these proportions are shown in Supplemental Figure S8. Error bars in panel *A* show the 95% quantiles from 10,000 bootstrap replicates in which differences in 1 − ρ between mel-mel and sim-sech as well as between sim-sech and mel-sim were calculated for each bootstrap replicate. The significance of the observed deviation from zero was determined by comparing the observed value to the distribution of bootstrap values. In panels *B* and *D*, significance was determined using Fisher’s exact tests. Significance of each comparison: (*) *P* ≤ 0.05, (**) *P* ≤ 0.001, (***) *P* ≤ 1 × 10^−4^.

Measures of relative gene expression derived from these mapped and normalized RNA-seq data correlated well with estimates of relative gene expression derived from independent pyrosequencing experiments (Supplemental Fig. S2A; Ahmadian et al. 2000). Genome-wide, expression levels between F_1_ hybrids from reciprocal crosses were also highly correlated (Fig. 2A; Supplemental Fig. S3). Despite this similarity, Fisher’s exact tests (FETs) with a false discovery rate (FDR) of 0.05 identified significant expression differences between reciprocal hybrids for 26%–49% of individual genes (Fig. 2B). Most of these significant expression differences were small in magnitude (median expression difference = 1.20- to 1.25-fold) (Supplemental Fig. S4), however, they reflect the sensitivity of the Fisher’s exact test for detecting differences in relative expression from RNA-seq data when read counts are high. These differences in expression between hybrids from reciprocal crosses provide a conservative baseline for expression differences detected in the mel-mel, sim-sech, and mel-sim comparisons because they include variance from technical and biological replication as well as parent-of-origin effects.

### Evolution of expression differences

To determine the tempo of regulatory divergence, we compared total expression levels in the mel-mel, sim-sech, and mel-sim comparisons for the set of 7587 genes in Data set 1 described above. First, we analyzed overall expression divergence (1 − Spearman’s ρ, see Methods) and found that it increased consistently and significantly with divergence time (Fig. 2A; Supplemental Fig. S5). We then used FETs to compare expression levels for individual genes and determine whether the increased overall expression divergence resulted from more genes with divergent expression or more divergent expression of similar numbers of genes. Surprisingly, we found that the proportion of genes with significant expression differences did not increase consistently with divergence time (Fig. 2B), suggesting that increasing magnitudes of expression differences rather than increasing numbers of genes with divergent expression drive the overall increase in expression differences with divergence time observed.

We also examined the evolutionary trajectories of individual genes by assigning each of the 7587 genes in Data set 1 to one of nine classes depending on whether its expression difference increased, decreased, or remained similar between mel-mel and sim-sech and between sim-sech and mel-sim. Expression differences less than 1.25-fold were considered similar for this analysis to minimize the impact of small but statistically significant expression differences (Supplemental Fig. S6). Despite observing that expression differences increased with divergence time on a genomic scale (Fig. 2A), this pattern was only seen for 2% of individual genes (Fig. 2C, class I). Expression differences of similar magnitude in all three comparisons were much more common (43% of all genes examined) and tended to be small in magnitude (median expression difference = 1.18-fold) (Fig. 2C, class II). The remaining 55% of genes fell into one of seven categories in which two of the three comparisons showed similar expression differences (Fig. 2C, class III). Interestingly, nearly half (45%) of such genes showed similar expression differences in mel-mel and mel-sim but larger or smaller expression differences in the sim-sech comparison (Fig. 2C, IIIc and IIId), which has an intermediate divergence time.

### Evolution of regulatory incompatibilities

Divergence of the regulatory networks controlling gene expression can cause misexpression in F_1_ hybrids that can contribute to speciation (Meiklejohn et al. 2003; Michalak and Noor 2004; Ranz et al. 2004; Haerty and Singh 2006; Moehring et al. 2007; Maheshwari and Barbash 2012). This can occur, for example, when proteins and/or DNA with sequence-specific interactions coevolve such that divergent alleles of the interacting molecules do not function properly together in F_1_ hybrids. To determine the rate at which misexpression resulting from such regulatory incompatibilities evolves, we compared expression levels in mel-mel, sim-sech, and mel-sim F_1_ hybrids to expression levels in the corresponding parental genotypes. We found that overall expression differences between parents and F_1_ hybrids increased with divergence time, most dramatically in the mel-sim comparisons (Fig. 2A; Supplemental Fig. S7). A similar increase was seen in the proportion of genes showing misexpression in F_1_ hybrids (Fig. 2B). The much more extensive misexpression seen in mel-sim F_1_ hybrids compared with mel-mel or sim-sech F_1_ hybrids is consistent with mel-sim F_1_ hybrid females having morphological defects that cause sterility (Dickinson et al. 1984) and mel-mel and sim-sech F_1_ hybrid females being completely fertile (Lachaise et al. 1986; Hollocher et al. 1997).

To further investigate the inheritance of gene expression levels and how inheritance patterns change over evolutionary time, we considered each gene separately and classified its expression in F_1_ hybrids as dominant, additive, misexpressed (i.e., over- or under-dominant), or similar (Supplemental Fig. S8). To minimize the impact of small but statistically significant expression differences on this analysis (Supplemental Fig. S9), we considered expression similar between genotypes if the expression difference was less than 1.25-fold. In the mel-mel F_1_ hybrids, we found that 7% of genes showed additivity, 14% showed misexpression, and 43% showed dominant inheritance. The remaining 36% of genes showed similar expression in both strains of *D. melanogaster* and in their F_1_ hybrids. The proportions of genes with additive and dominant inheritance decreased consistently with divergence time, whereas the proportion of genes showing misexpression increased dramatically with divergence time (Fig. 2D).

### Using allele-specific RNA-seq reads to study regulatory evolution

Differences in gene expression can be caused by changes in *cis*- and/or *trans*-regulation. Understanding the relative contribution of these two types of changes is critical for understanding the mode of regulatory evolution (Gordon and Ruvinsky 2012). To separate the effects of *cis*- and *trans*-regulatory divergence, we analyzed allele-specific expression in F_1_ hybrids and contrasted it with comparable measures of total expression differences between parental genotypes derived from allele-specific reads in “mixed parental” samples. These mixed parental samples were constructed in silico by combining equal numbers of mapped RNA-seq reads from each parental genotype and subjected to the same bioinformatic analysis as the reads from F_1_ hybrids. Expression differences between alleles in F_1_ hybrids were attributed to *cis*-regulatory differences, and differences in relative expression between parental genotypes that were not explained by differences in *cis*-regulatory activity were attributed to *trans*-regulatory divergence (Wittkopp et al. 2004).

For each F_1_ hybrid and mixed parent sample, RNA-seq reads that aligned perfectly and uniquely to one parental genome but not the other were considered allele-specific. Genes with low confidence allele assignments (see Supplemental Material), fewer than 20 total allele-specific reads, or expression consistent with genomic imprinting in any comparison were excluded from analysis (Supplemental Table S3). For each of the remaining 4851 genes, differences in the number of allele-specific reads among comparisons were eliminated by using hypergeometric sampling to produce a data set with the same number of allele-specific reads in all comparisons (Data set 2). Measures of relative total expression derived from allele-specific reads in the mixed parental samples were strongly correlated with measures of relative total expression derived from the full RNA-seq data set (Supplemental Fig. S10) and pyrosequencing (Supplemental Fig. S2B). Relative allele-specific expression in F_1_ hybrids also showed a strong correlation between the RNA-seq and pyrosequencing data (Supplemental Fig. S2C) and was similar in F_1_ hybrids from reciprocal crosses (Supplemental Fig. S11). In the analyses described below, hybrids from reciprocal crosses were considered separately, with results from one hybrid for each comparison presented in the main text and results from the other hybrid presented in the Supplemental Material. With few exceptions (noted below), results were similar between reciprocal hybrids.

### Evolution of *cis*- and *trans*-regulation

**Figure 3.**
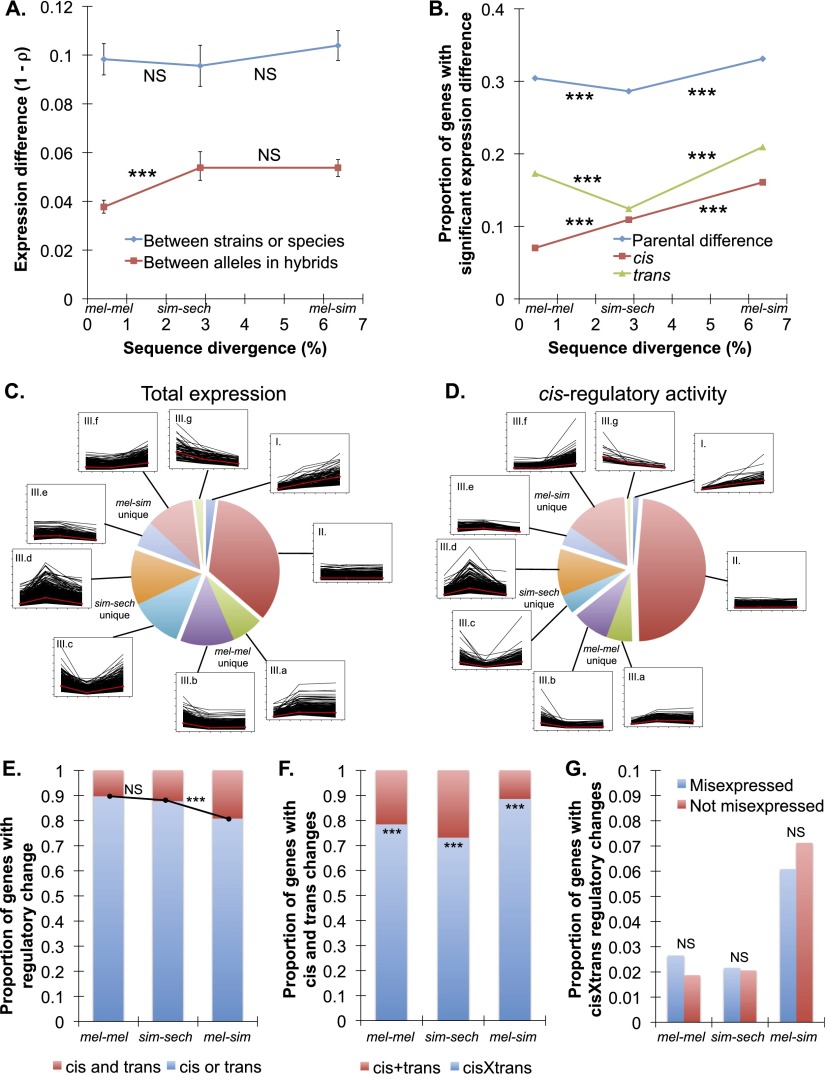
Evolution of *cis*- and *trans*-regulation. (*A*) Overall differences (1 − ρ) in total expression between genotypes (blue) and allele-specific expression in F_1_ hybrids (red) are shown for each comparison, with data used for these calculations shown in Supplemental Figure S13. Relative allelic expression in F_1_ hybrids provides a readout of relative *cis*-regulatory activity. (*B*) For each comparison, the proportions of genes with evidence of significant differences in total expression (blue), *cis*-regulation (red), and *trans*-regulation (green) are shown. Data used to determine these proportions are shown in Supplemental Figure S16. Significance tests used to identify differences in *trans*-regulation had a different power than those used to identify differences in total expression and *cis*-regulation, thus only the evolutionary trends, not the proportions of significant genes, should be compared among these classes. Power was comparable, however, in the tests for differences in total expression and relative *cis*-regulatory activity summarized in this figure. (*C*,*D*) The line plots show expression differences (*C*) and differences in relative *cis*-regulatory activity (*D*) for individual genes in the mel-mel, sim-sech, and mel-sim comparisons plotted according to divergence time, with the 4851 genes included in Data set 2 classified into nine groups depending on whether they showed increased, decreased, or similar expression differences between mel-mel and sim-sech and between sim-sech and mel-sim. The red line in each plot shows the median expression difference for genes in that class for each comparison. The pie charts show the relative frequency of genes in each class. (*E*) The proportion of genes with evidence of significant *cis*- and *trans*-regulatory changes (red) is compared with the proportion of genes with evidence of *cis*- or *trans*-regulatory changes (blue). (*F*) For genes with evidence of both *cis*- and *trans*-regulatory changes, the frequency of genes with *cis*- and *trans*-regulatory changes affecting gene expression in the same (“*cis* + *trans*,” red) and opposite (“*cis* × *trans*,” blue) directions are compared. (*G*) The relative frequencies of genes with *cis*- and *trans*-regulatory changes in opposite directions that do (blue) and do not (red) show evidence of misexpression in F_1_ hybrids are compared. Error bars in panel *A* show the 95% quantiles from 10,000 bootstrap replicates in which differences in 1 − ρ between mel-mel and sim-sech as well as between sim-sech and mel-sim were calculated for each bootstrap replicate. The significance of the observed deviation from zero was determined by comparing the observed value to the distribution of bootstrap values. Significance was determined using Fisher’s exact tests in panels *B*, *E*, and *G* and using binomial exact tests in panel *F*. Significance of each comparison: (NS) Nonsignificant, *P* > 0.05; (*) *P* ≤ 0.05; (**) *P* ≤ 0.001; (***) *P* ≤ 1 × 10^−4^. Comparable analyses for reciprocal hybrids are shown in Supplemental Figures S12, S15.

To determine the rate of *cis*-regulatory divergence and compare it with the rate of total expression divergence for the same genes, we contrasted overall differences in relative allelic abundance between the F_1_ hybrid and mixed parental samples for the 4851 genes deemed suitable for measuring allele-specific expression (Data set 2). Compared with the 7587 genes discussed above (Data set 1), this set of genes showed more similar levels of overall expression differences among the three comparisons (Fig. 3A; Supplemental Figs. S12A, S13A–C), resulting from increased expression divergence in mel-mel and sim-sech in Data set 2 relative to Data set 1 (Supplemental Fig. S14). Despite this similarity in total expression differences among comparisons, we found that *cis*-regulatory differences were greater between than within species, with similar differences in relative *cis*-regulatory activity observed in sim-sech and mel-sim (Fig. 3A; Supplemental Figs. S12A, S13D–I). Comparing the proportions of genes with statistically significant differences in total expression and *cis*-regulatory activity showed a similar pattern, except that the proportion of genes with evidence of a *cis*-regulatory difference increased consistently and significantly with divergence time (Fig. 3B; Supplemental Fig. S12B). This suggests that the greater overall *cis*-regulatory divergence observed in the sim-sech comparison for these 4851 genes results from large differences in relative *cis*-regulatory activity for some genes rather than an excess of genes with divergent *cis*-regulatory activity.

We also compared the evolutionary trajectories of individual genes for total expression differences (Fig. 3C) and relative *cis*-regulatory activity (Fig. 3D; Supplemental Fig. S15) by dividing the 4851 genes in Data set 2 into the same nine classes described above for Data set 1 (Fig. 2C). Compared with total expression, we found that more genes showed consistent and small (median = 1.16-fold) differences in relative *cis*-regulatory activity in all three comparisons (Fig. 3C, II, 3D, II). We also observed more genes with unique differences in *cis*-regulatory activity in sim-sech (Fig. 3D, IIIc,d) and mel-sim (Fig. 3D, IIIe,f) that were greater in these comparisons than the other two comparisons. In other words, genes with a similar difference in *cis*-regulatory activity in mel-mel and mel-sim but not sim-sech were more likely to show increased than decreased divergence in sim-sech relative to the other two comparisons. Such asymmetry was much less pronounced for levels of total expression (Fig. 3C), suggesting that *trans*-acting changes have compensated for differences in *cis*-regulatory activity in many cases.

Differences between divergent *cis*-regulatory activity and total gene expression are caused by the divergence of *trans*-regulatory factors. We found that significantly more genes showed evidence of *trans*-regulatory differences in the mel-mel and mel-sim comparisons than in the sim-sech comparison (Fig. 3B; Supplemental Fig. S12B). This suggests that *cis*-regulatory divergence accounts for a larger proportion of overall expression divergence in sim-sech than in mel-mel or mel-sim. Consistent with this inference, a regression analysis showed that *cis*-regulatory differences explained more of the expression differences between *D. simulans* and *D. sechellia* than between either of the other two pairs of genotypes (Supplemental Fig. S16).

As overall sequence divergence increases, the number of loci with variation affecting expression of each gene is also expected to increase. Consistent with this expectation, we found that the proportion of genes with regulatory changes showing evidence of both *cis*- and *trans*-regulatory changes increased with divergence time, although the increase between the mel-mel and sim-sech comparisons was only statistically significant for one of the two hybrids (Fig. 3E; Supplemental Fig. S12C). For the majority of these genes, the *cis*- and *trans*-regulatory changes favored expression of alternative alleles (Fig. 3F; Supplemental Fig. S12D), suggesting that stabilizing selection has favored regulatory mutations that reduce expression differences. As described above, this type of developmental systems drift (True and Haag 2001) is thought to cause misexpression in F_1_ hybrids (Michalak and Noor 2004; Ranz et al. 2004; Landry et al. 2005; McManus et al. 2010; Barrière et al. 2012; Maheshwari and Barbash 2012). The frequency of genes with compensatory *cis*- and *trans*-regulatory changes did not increase steadily with divergence time; however, *cis*- and *trans*-regulatory changes favoring expression of opposite alleles were observed least often in the sim-sech comparison (Fig. 3F; Supplemental Fig. S12D). Contrary to prior studies (Landry et al. 2005; Tirosh et al. 2009; McManus et al. 2010), we found that genes affected by *cis*- and *trans*-regulatory changes with opposing effects on total expression levels were not more likely to show misexpression in F_1_ hybrids (Fig. 3G; Supplemental Fig. S12E).

**Figure 4.**
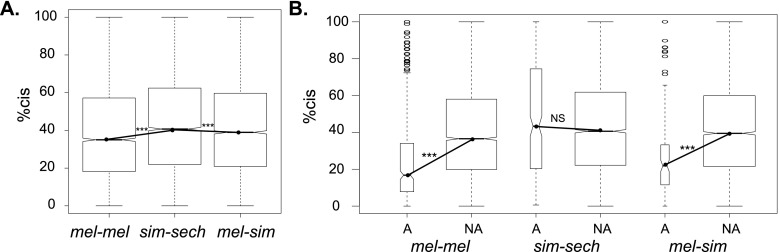
Effects of *cis*-regulatory divergence. (*A*) The percentage of total regulatory divergence attributable to *cis*-regulatory divergence (% *cis*) is shown for the mel-mel, sim-sech, and mel-sim comparisons. (*B*) % *cis* is compared for sets of genes showing additive (“A”) and nonadditive (“NA” [dominant or misexpression]) inheritance for each comparison. In all panels, notched box plots show the full range of values as well as the 25th, 50th, and 75th percentiles. Within both panels, the widths of the boxes are proportional to the number of genes represented. Statistical significance of differences between median values connected with solid lines was determined using Mann-Whitney *U*-tests. (*) *P* ≤ 0.05, (**) *P* ≤ 0.001, (***) *P* ≤ 1 × 10^−4^. Comparable analyses for reciprocal hybrids are shown in Supplemental Figure S17.

To determine how the relative effects of *cis*- and *trans*-regulatory changes vary with divergence time, we calculated the percentage of total regulatory divergence attributable to *cis*-regulatory changes for each gene. This value is referred to as “percent *cis*” (% *cis*), and prior studies of flies (Wittkopp et al. 2008; McManus et al. 2010) and yeast (Emerson et al. 2010) found it to be larger between than within species. We also found that % *cis* was larger between than within species; however, in contrast to prior predictions (Wittkopp et al. 2008), % *cis* did not increase systematically with divergence time. Rather, it was largest for the sim-sech comparison with intermediate divergence time (Fig. 4A; Supplemental Fig. S17A). A correlation between % *cis* and total expression divergence for individual genes was previously reported between *D. melanogaster* and *D. sechellia* (McManus et al. 2010), but we did not observe this pattern for any of the three comparisons (Supplemental Fig. S17B–G). Finally, two prior studies (Lemos et al. 2008; McManus et al. 2010) reported that % *cis* was higher for genes showing additive than nonadditive (i.e., dominant, over-dominant, or under-dominant) inheritance. We observed this relationship only for the comparison of *D. simulans* and *D. sechellia* in one hybrid (Fig. 4B; Supplemental Fig. S17H), suggesting that it is also not a general feature of regulatory evolution.

## Discussion

Researchers have been comparing genomic patterns of expression divergence among species for over a decade using microarrays, but sequence divergence between microarray probes and RNA samples often complicates comparisons among species and differences in normalization and statistical analyses can complicate comparisons among studies. Here, we use RNA-seq data to determine the tempo and mode of regulatory evolution among four divergent strains and species of *Drosophila*. This technique is better suited for interspecific comparisons than microarrays because it uses full sequence information instead of hybridization signals to determine gene expression levels, allowing more direct comparisons among species and studies.

**Figure 5.**
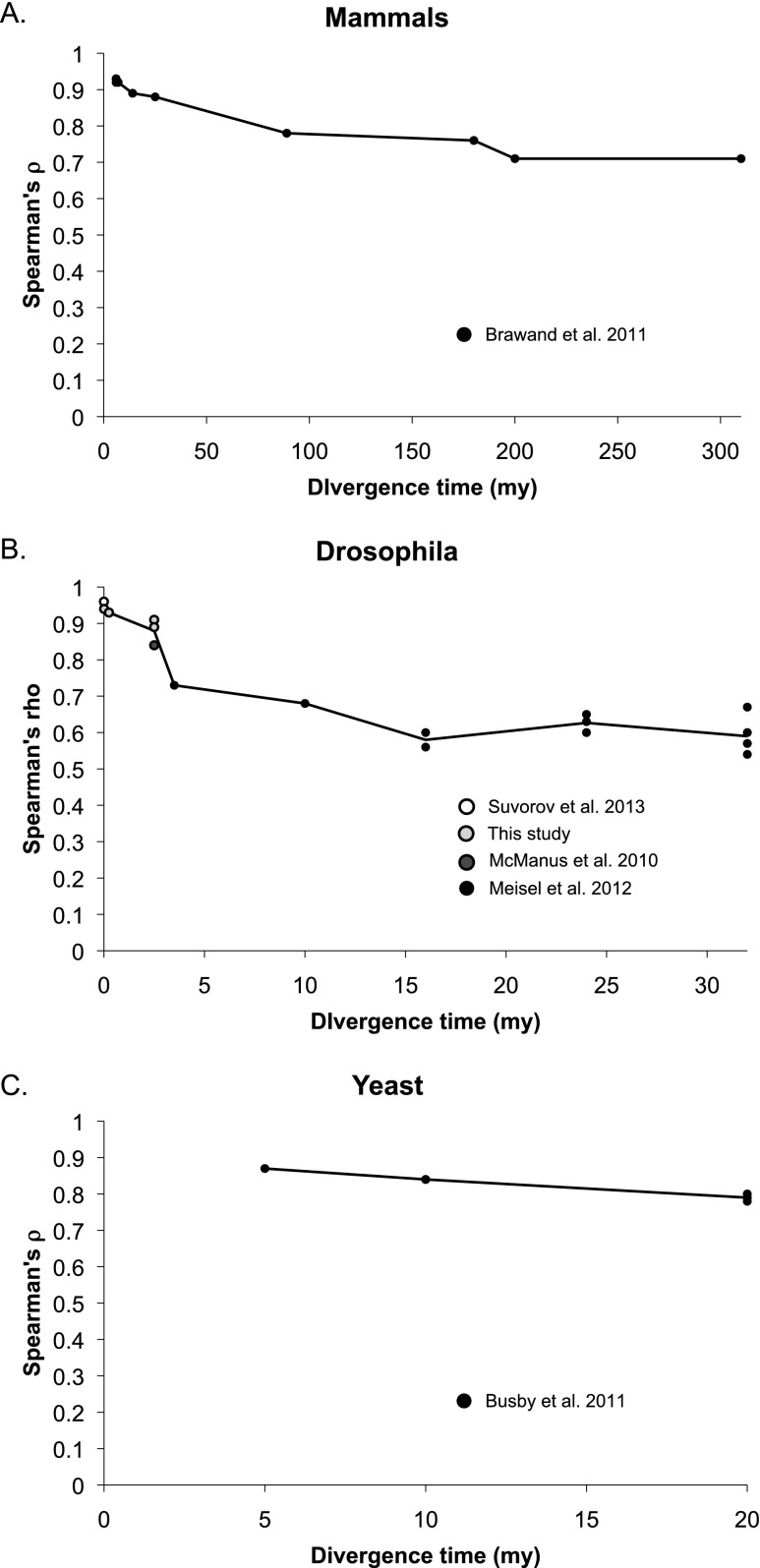
Expression divergence in mammals, *Drosophila*, and yeast. (*A*) Expression similarity (Spearman’s ρ) was calculated using RNA-seq data from kidneys published in Brawand et al. (2011) comparing human samples with those of eight other mammalian species and one bird. We chose to analyze the data from kidneys because they were the most representative of all the tissues examined (excluding testes). Divergence times in millions of years are as reported in Brawand et al. (2011). (*B*) Expression similarity (Spearman’s ρ) was calculated for data described in this paper (light gray circles) as well as data published in Suvorov et al. (2013) (open circles), McManus et al. (2010) (gray circles), and Meisel et al. (2012) (black circles). Divergence times for mel-mel, sim-sech, and mel-sim are as described in Figure 1A. For all other comparisons, estimated divergence times from Obbard et al. (2012) were used. (*C*) Expression similarity (Spearman’s ρ) was calculated using the data reported in Busby et al. (2011) for all pairwise comparisons of four yeast species. Divergence times for these species are from Kellis et al. (2003). In all three cases, the black line connects the average value of ρ for each divergence time sampled.

RNA-seq was also recently used to compare expression levels in six different tissues among nine mammalian species and a bird (Brawand et al. 2011). Using Spearman’s rank correlation coefficient ρ to compare overall expression differences in each pair of species, this study showed that expression similarity decreased quickly over shorter divergence times and then slowed. Patterns of expression divergence were strikingly similar among RNA samples from brain (cerebral cortex or whole brain without cerebellum), cerebellum, heart, kidney, and liver, with accelerated expression divergence in RNA samples from testes (Brawand et al. 2011). By combining our data with data from three previous studies (McManus et al. 2010; Meisel et al. 2012; Suvorov et al. 2013), we found that expression divergence among *Drosophila* species showed a similar pattern to that of mammals, but on a different timescale (Fig. 5A,B). The *Drosophila* data showed greater expression divergence (lower values of ρ) than the mammalian data, which could be due to differences in tissue size among *Drosophila* species given that whole bodies rather than single tissues were used to generate these data. RNA-seq has also been used to compare expression divergence among four species of yeast (Busby et al. 2011), but it is difficult to compare the tempo in yeast to that of *Drosophila* and mammals because only three divergence time points were sampled (Fig. 5C).

For each gene, interspecific expression differences can be caused by *cis*- and/or *trans*-regulatory changes. When F_1_ hybrids can be made between species, measures of allele-specific expression can be used to disentangle the net effects of these two types of changes (Wittkopp et al. 2004). Such analyses have been reported for closely related pairs of strains or species in yeast (Tirosh et al. 2009; Emerson et al. 2010; Schaefke et al. 2013), flies (Graze et al. 2009; McManus et al. 2010; Coolon et al. 2012), plants (Shi et al. 2012; Bell et al. 2013), fishes (Murata et al. 2012; Shen et al. 2012), and mice (Goncalves et al. 2012). To the best of our knowledge, this is the first genomic study collecting data on *cis*- and *trans*-regulatory divergence for more than one pair of genotypes. As such, it provided unprecedented insight into the rate at which *cis*- and *trans*-regulatory changes evolve and allowed us to better assess the generality of relationships reported in other studies.

### Compensatory *cis*- and *trans*-regulatory changes are common

We found that the number of genes with evidence for *cis*-regulatory divergence increased linearly with divergence time, but the number of genes with differences in total expression did not (Fig. 3B; Supplemental Fig. S12D). This suggests that *trans*-regulatory factors might often compensate for *cis*-regulatory differences at the level of total gene expression, either by fixing compensatory *trans*-regulatory variants or by feedback mechanisms affecting availability or activity of *trans*-acting factors (McManus et al. 2014). Consistent with this interpretation, *cis*- and *trans*-acting changes affecting expression of the same gene had opposite effects on expression levels 79%, 73%, and 87% of the time in the mel-mel, sim-sech, and mel-sim comparisons, respectively (Fig. 3F). The exponential accumulation of genes that are misexpressed in F_1_ hybrids (Fig. 2D) is also consistent with compensatory changes playing an important role in maintaining gene expression levels over evolutionary time (Landry et al. 2005). Such compensation can result from stabilizing selection acting to maintain similar expression levels in the face of new mutations, and has been seen not only in flies, but also in yeast (Tirosh et al. 2009), mice (Goncalves et al. 2012), and plants (Shi et al. 2012).

Compensation for *cis*-regulatory divergence resulting from the fixation of *trans*-acting changes could evolve by fixing *cis*-acting mutations first and then compensating *trans*-acting mutations, or vice versa. We favor the latter model because *trans*-acting mutations appear to arise more frequently than *cis*-acting mutations for individual genes (Gruber et al. 2012) and most *trans*-acting mutations that compensate for *cis*-regulatory divergence of one gene are expected to have deleterious pleiotropic effects on expression of other genes (Wray et al. 2003; Carroll 2008; Stern and Orgogozo 2008). Goncalves et al. (2012) favored a similar explanation for the extensive compensatory *cis*- and *trans*-regulatory changes they observed between strains of mice. An example of such *trans*-regulatory divergence subsequently compensated for by *cis*-regulatory changes has been described in yeast (Kuo et al. 2010). Regardless of which type of regulatory mutation is usually fixed first, it is clear that the regulatory networks controlling gene expression evolve more rapidly than the output from these networks.

### Relative impact of selection and drift on regulatory evolution

A common goal for comparative studies of gene expression is identifying the selective and nonselective forces responsible for patterns of divergence and conservation, but this is not straightforward (Gilad et al. 2006b; Fay and Wittkopp 2008; Emerson et al. 2010). Without the biological replication needed to make statistically robust inferences based on alternative evolutionary models (e.g., Rifkin et al. 2003; Fay and Wittkopp 2008; Bedford and Hartl 2009; Brawand et al. 2011), we can only make speculative statements about the evolutionary processes responsible for each of the nine different trajectories of expression divergence we observed (Figs. 2C, 3C,D). For example, genes with similar (and typically small) expression differences in all three comparisons (class II in Figs. 2C and 3C,D) may either have low mutation-drift variance or be subject primarily to stabilizing selection. This is the most abundant class of genes for both total expression and *cis*-regulatory activity with 43% and 34% of genes showing this pattern for total expression in Data sets 1 and 2, respectively, and 48% of genes showing this pattern for differences in *cis*-regulatory activity in Data set 2. This is consistent with prior work suggesting that stabilizing selection has had a larger impact on the evolution of gene expression than genetic drift (Hsieh et al. 2003; Rifkin et al. 2003; Lemos et al. 2005; Gilad et al. 2006a; Xing et al. 2007; Kalinka et al. 2010). Indeed, <2.2% of genes in each comparison showed the increasing differences in total expression and/or *cis*-regulatory activity with divergence time (class I in Figs. 2C, 3C,D, and Supplemental Fig. S15) that are expected when expression evolves primarily due to genetic drift (Khaitovich et al. 2004; Gilad et al. 2006a). The remaining genes fell into one of seven categories consistent with variable selection pressures among lineages (class III in Figs. 2C and 3C,D).

### Lineage-specific regulatory changes in *D. sechellia*

Gene-specific patterns of total expression divergence consistent with lineage-specific selection were more abundant in sim-sech than mel-mel or mel-sim for both Data sets 1 and 2 despite the sim-sech comparison having an intermediate divergence time (Figs. 2C, 3C). This is consistent with *D. sechellia* being an island endemic species with a small effective population size that has evolved many novel phenotypes relative to *D. melanogaster* and *D. simulans* (Orgogozo and Stern 2009), including adaptation to a new host plant (Jones 2005). As a consequence of this evolutionary history, *D. sechellia* might have fixed more deleterious mutations than the other two species by drift as well as more adaptive substitutions by positive selection. We observed an apparent excess of *cis*-regulatory divergence between *D. simulans* and *D. sechellia* (Figs. 3A, 4A; Supplemental Fig. S16) that we believe is more likely to result from positive selection than drift because (1) *trans*-acting variation contributes more than *cis*-acting variation to polymorphic expression within species (Lemos et al. 2008; Wittkopp et al. 2008; Emerson et al. 2010), suggesting that drift is more likely to fix *trans*-acting than *cis*-acting variants; (2) *cis*- and *trans*-regulatory changes affecting expression of the same gene were most likely to act in the same direction in the sim-sech comparison (Fig. 3F), which is consistent with positive, directional selection; and (3) simulation studies have shown that *cis*-regulatory divergence is more likely to be driven by natural selection than *trans*-regulatory divergence (Emerson et al. 2010). These results emphasize the importance of considering not only divergence time, but also the demographic and ecological history of individual species when studying the tempo and mode of evolution.

## Methods

### Fly strains, rearing, and collections

Four *Drosophila* genotypes were used for this study: the *D. melanogaster* North American zhr strain [full genotype: XYS.YL.Df(1)Zhr] (Sawamura and Yamamoto 1993; Ferree and Barbash 2009), the *D. melanogaster* Zimbabwean isofemale strain z30 (Begun and Aquadro 1993; Wu et al. 1995), the sequenced *D. sechellia* strain (droSec1 [14021-0428.25]), and an isofemale strain of *D. simulans* (Tsimbazaza) that mates well with *D. melanogaster* (Hollocher et al. 2000). All flies were reared on cornmeal medium using a 16:8 light:dark cycle at 20°C. Just prior to the start of the experiment, all strains were subjected to 10 generations of sibling pair matings to reduce genome-wide heterozygosity, followed by three generations of population expansion to generate the quantity of flies needed for crosses. For each cross between strains of *D. melanogaster*, 10 vials were set up with three female and three male flies each. For each interspecific cross, 30 vials were set up with three female and three male flies each. Virgin female progeny were allowed to mate from the time of eclosion to 3 d post-eclosion, then males and females were separated and females aged to 7–10 d post-eclosion. All flies were collected between 9 and 10 am to minimize the effects of circadian rhythm and snap-frozen in liquid nitrogen.

### Sample preparation and sequencing

For each genotype analyzed, a pool of 20 female flies was used for total RNA extraction with TRIzol reagent according to manufacturer instructions (Invitrogen). This incorporates variation from biological replication into a single sample. Prior work has shown that expression for most genes is similar among replicate pools constructed in this way (Wittkopp et al. 2004, 2008; Coolon et al. 2012). Genomic DNA (gDNA) was extracted from a separate pool of 20 flies for each genotype using the DNeasy Blood & Tissue Kit (Qiagen). Illumina sequencing libraries for RNA-seq were prepared as previously reported (McManus et al. 2010; Coolon et al. 2012). Briefly, 10 µg of total RNA from each sample was treated with DNase I (Invitrogen) followed by poly(A)^+^ selection using Dynal magnetic beads (Invitrogen). Poly(A)^+^ RNA was fragmented using RNA fragmentation reagent (Ambion) before cDNA synthesis. Double-stranded cDNA was produced using random hexamers and SuperScript II reverse transcriptase (Invitrogen). cDNA was run on a 2% agarose gel and the region corresponding to ∼300-bp fragments was extracted. The size-selected double-stranded cDNA extracted from this gel slice was used in the Paired-End Genomic DNA Library Preparation Kit (Illumina) according to manufacturer’s recommendations. For the gDNA sequencing libraries, 10 µg of gDNA was used with the Paired-End Genomic DNA Library Preparation Kit (Illumina), following manufacturer’s recommendations. Each cDNA and gDNA library was subjected to a full lane of paired-end sequencing on an Illumina Genome Analyzer IIx using 76 cycles. On average, 24 million 76-bp, paired-end sequence reads were generated from each sequencing library (Supplemental Table S1). The zhr gDNA sample was also sequenced from a single end on an additional lane for 76 cycles per read. Images were analyzed using the Firecrest and Bustard modules to generate sequence and quality scores for each read.

### Resequencing, genome assembly, and sequence divergence

Using the gDNA sequences, we constructed a strain-specific genome sequence for each genotype as described in the Supplemental Material. To determine percent sequence divergence in each comparison (mel-mel, sim-sec, mel-sim), we created reverse chain files to liftOver coordinates from *D. melanogaster* dm3 space to each of the other strain or species genomic space (zhr, z30, Tsimbazaza, droSec1) using the chainSwap utility from the UCSC Genome Browser (Kent et al. 2002). Using these chain files, we converted the dm3 genomic coordinates for each exon used for quantification in this study into their respective strain- or species-specific genomic coordinates. Using these coordinates, sequences for each exon were extracted from each strain- or species-specific genome. These sequences were aligned in pairs using Fast Statistical Alignment (FSA) (Bradley et al. 2009), and the number of divergent sites per gene was determined using custom perl scripts (pairwise_aln_FSA.pl, compare_pairwise.pl, seq_div_from_set.pl). Strain-specific genomes and chain files are provided in Supplemental File 1, and all custom perl scripts are included in Supplemental File 2.

### Mapping sequencing reads to genes and alleles

We built a bioinformatics pipeline to measure total and allele-specific expression from Illumina sequencing outputs similar to those reported previously (McManus et al. 2010; Coolon et al. 2012). This pipeline, as well as the pyrosequencing methods used to validate measures of total and allele-specific expression derived from this pipeline, is described in the Supplemental Material.

### Normalizing RNA-seq read counts among comparisons

Different numbers of sequence reads were recovered for each of the 10 cDNA libraries sequenced. These differences in read counts caused the Fisher’s exact tests used to identify significant changes in gene expression between pairs of genotypes to have differences in power among the mel-mel, sim-sech, and mel-sim comparisons. To equalize power in all three comparisons, we considered exactly 12,704,991 mapped reads from each RNA-seq data set by down-sampling mapped reads randomly without replacement in all but the *D. sechellia* data set, which already had exactly 12,704,991 mapped reads (Supplemental Table S2). A similar down-sampling strategy was recently used to investigate the power of different bioinformatic tools for identifying expression differences (Rapaport et al. 2013). We then excluded genes with fewer than 20 reads in any of the RNA-seq data sets, resulting in the same 7587 “expressed” genes being analyzed in each comparison (Supplemental Table S2). Simulations confirmed that a larger data set down-sampled in this way has the same power to detect significant expression differences with a Fisher’s exact test as a data set originally collected at the smaller sample size (data not shown). The exact data analyzed are provided in Supplemental Material as Data set 1.

### Comparing total expression among genotypes

Spearman’s correlation coefficients (ρ) were used to measure overall expression differences between pairs of genotypes, following Brawand et al. (2011) and Meisel et al. (2012). Unlike Pearson’s r, Spearman’s ρ makes no assumptions about normality, linearity, or homoscedasticity. It is also less sensitive to outliers. Bootstrapping was used to test for statistically significant differences in ρ between mel-mel and sim-sech and between sim-sech and mel-sim by sampling with replacement 7587 gene-specific read counts from the observed 7587 genes 10,000 times using R, calculating ρ in each case, and determining the 2.5% and 97.5% percentiles. Significant differences were inferred when these 95% quantiles did not overlap.

We also tested for significant differences in expression level of individual genes by comparing the number of reads mapping to the focal gene to the number of reads mapping to the other 7586 genes between parental types, between reciprocal hybrids, and between each hybrid and parent using Fisher’s exact tests with a null hypothesis of equal expression in both samples. This test was used instead of other methods for detecting differential expression because it recovers a similar proportion of true positives with fewer false positives without requiring replicates (Tarazona et al. 2011). Fisher’s exact tests were also used to test for significant differences in the proportion of genes with significant differences between mel-mel and sim-sech and between sim-sech and mel-sim.

### Inferring the mode of inheritance

To determine the mode of inheritance for each gene in each comparison, we followed the logic outlined in Gibson et al. (2004) and used previously for RNA-seq data in McManus et al. (2010). Using a 1.25-fold expression difference cutoff and total expression levels in the F_1_ hybrids and corresponding parental genotypes, we classified each gene as either “similar,” “additive,” “parent 1 dominant,” “parent 2 dominant,” “under-dominant,” or “over-dominant.” Dominant inheritance was inferred when total expression in the F_1_ hybrid was similar to expression in one of the parental genotypes but different from the other parental genotype. Such genes were classified as either “parent 1 dominant” or “parent 2 dominant” depending on which parent the F_1_ hybrid resembled. Additive inheritance was inferred when F_1_ hybrid expression was different from, and intermediate to, both parents; and misexpression was inferred when the total expression in the F_1_ hybrid was different from both parental genotypes and greater than (over-dominant) or less than (under-dominant) the more extreme parental expression level. Genes with similar expression in both parents and F_1_ hybrids were classified as similar. Fisher’s exact tests were used to test for significant differences in the proportion of genes in each category between mel-mel and sim-sech and between sim-sech and mel-sim.

### Normalizing allele-specific RNA-seq read counts among comparisons

To equalize power when testing for *cis*-regulatory divergence in mel-mel, sim-sech, and mel-sim, as well as when comparing tests for *cis*-regulatory and total expression divergence, we created a second data set with the same number of allele-specific reads for each gene in all comparisons. This data set was constructed by (1) combining the equal numbers of mapped reads for each genotype used in the first data set to make a “mixed parental” sample for each comparison (e.g., reads from zhr and z30 were combined for the mel-mel comparison); (2) counting allele-specific reads (i.e., reads that mapped perfectly and uniquely to only one of the parental genomes) in all mixed parental and F_1_ hybrid samples; and (3) equalizing allele-specific read counts for each gene in all mixed parental and hybrid samples by identifying the sample with the fewest allele-specific reads for that gene and using hypergeometric sampling of the observed allele-specific read counts to randomly reduce the number of allele-specific reads considered in each of the other samples. Simulations confirmed that this down-sampling approach produced data sets with the same power to detect significant expression differences with Fisher’s exact tests as data sets originally collected at the smaller sample sizes (data not shown), and a similar method was recently used for allele-specific RNA-seq data from humans (Lappalainen et al. 2013).

Prior to analysis, genes with low confidence allele-assignments in the mel-mel, sim-sech, or mel-sim comparisons, defined as having >10% of the mapped reads from one parent aligned solely to the genome of the other parent, were excluded. Genes with less than 20 total allele-specific reads (allele 1 + allele 2 < 20) in any mixed parental or hybrid sample were also excluded from all comparisons; this threshold was based on prior theoretical and empirical work (Fontanillas et al. 2010; McManus et al. 2010). Finally, nine more genes were excluded because they showed significant differences in relative allelic expression between reciprocal hybrids using Fisher’s exact tests with a null hypothesis of equal expression and an FDR of 0.05. Such differences in relative allelic expression can result from parent-of-origin effects such as mitochondrial inheritance or genomic imprinting; imprinting seems rarely, if ever, responsible for this pattern of expression in *Drosophila*, however (Wittkopp et al. 2006, 2008; Coolon et al. 2012). After applying these filters, 4851 genes were deemed suitable for allele-specific analysis in all comparisons, with most of the genes excluded from this data set because they had too few allele-specific reads in the mel-mel comparison (Supplemental Table S3).

Mitochondrial genes were excluded from our allele-specific data set; however, allele assignments for F_1_ hybrid reads that mapped to mitochondrial genes were used as one metric to evaluate the reliability of our bioinformatic allele assignments. In the absence of sequencing and allele-assignment errors, all of these reads should map to the maternal allele. We found that 99.5% and 99.8% of reads from mitochondrial genes mapped to the maternal allele in F_1_ hybrids between *D. simulans* and *D. sechellia* and between *D. melanogaster* and *D. simulans*, respectively (Supplemental Table S4). Additional validation of allele assignments is described in the main text.

The exact data analyzed are provided in the Supplemental Material as Data set 2.

### Evaluating *cis*- and *trans*-regulatory changes

Spearman’s ρ was used to measure *cis*-regulatory divergence on a genomic scale in the mel-mel, sim-sech, and mel-sim comparisons by assessing the correlation between allele 1 and allele 2 read counts from F_1_ hybrids. It was also used to repeat the analysis of overall expression divergence in each comparison using the mixed parental samples. To test for statistically significant differences in ρ between mel-mel and sim-sech and between sim-sech and mel-sim, we used bootstrapping. Specifically, we sampled with replacement 4851 gene-specific read counts from the observed 4851 genes 10,000 times using R, calculated ρ in each case, and determined the 2.5% and 97.5% percentiles. Significant differences were inferred when these 95% quantiles did not overlap.

Binomial exact tests with a null hypothesis of equal expression were used to identify significant expression differences between genotypes in the mixed parental pools as well as significant differences in relative allelic expression in the F_1_ hybrid samples that indicate differences in relative *cis*-regulatory activity. An FDR of 5% was used to determine statistical significance despite the fact that the *P*-values produced by binomial exact tests when the null hypothesis is true are not uniformly distributed as assumed by the FDR correction for multiple tests (Skelly et al. 2011). This is because our simulations showed that the violation of this assumption had no effect on the number of genes called significant in this study (Supplemental Material). To test for the unequal allelic abundance between mixed parental and F_1_ hybrid samples that would indicate *trans*-regulatory divergence, we performed Fisher’s exact tests with a null hypothesis of equal expression by comparing read counts from genotype 1 and genotype 2 in the mixed parental sample to allele 1 and allele 2 in the corresponding F_1_ hybrid samples. Each gene in each comparison was classified as “conserved,” “all *cis*,” “all *trans*,” “*cis* + *trans*,” “*cis* × *trans*,” “compensatory,” or “ambiguous” based on the results of the Fisher’s and binomial exact tests using the criteria described in Supplemental Table S5. These same classifications were used previously in Landry et al. (2005) and (McManus et al. 2010). Fisher’s exact tests were also used to test for significant differences in the proportion of genes with significant differences between mel-mel and sim-sech and between sim-sech and mel-sim.

For each gene in each comparison, the total expression difference was calculated as log_2_(genotype 1 read count/genotype 2 read count) from the mixed parental sample, and the *cis*-regulatory difference (“*cis*”) was calculated as log_2_(allele 1 read count/allele 2 read count) from each of the F_1_ hybrid samples. The *trans*-regulatory difference (“*trans*”) for each gene in each comparison was calculated as the difference between the total expression and *cis*-regulatory differences: log_2_(genotype 1 read count/genotype 2 read count) − log_2_(allele 1 read count/allele 2 read count). % *cis* (proportion of total regulatory divergence attributable to *cis*-regulatory changes) was then calculated as [|cis|/(|cis| + |trans|)] × 100.

### Scripts and software used

All statistical analyses, down-sampling, and simulations were performed in R (version 2.12.2 or version 3.0.1, CRAN) (R Development Core Team 2008). This code includes the use of fisher.test for Fisher’s exact tests, binom.test for binomial exact tests, corr.test for Spearman’s ρ, sample to randomly down-sample mapped reads and simulate mapped read counts from a multivariate distribution, rhyper to randomly down-sample allele-specific read counts, rbinom to simulate allele-specific read counts. Custom perl and R scripts used in this work are included in Supplemental File 2.

## Data access

The sequencing data from this study have been submitted to the NCBI Sequence Read Archive (SRA; http://www.ncbi.nlm.nih.gov/sra) under accession numbers SRA052065 and SRP023274.
